# Describing the longitudinal course of major depression using Markov models: Data integration across three national surveys

**DOI:** 10.1186/1478-7954-3-11

**Published:** 2005-11-15

**Authors:** Scott B Patten, Robert C Lee

**Affiliations:** 1 Department of Community Health Sciences, University of Calgary, 3330 Hospital Drive NW, Calgary, Canada; 2 Health Technology Implementation Unit, Calgary Health Region. Foothills Medical Centre, South Tower, Room 602. 1403 29^th ^Street NW, Calgary, Canada; 3 Department of Psychiatry, University of Calgary, 1403 – 29 Street NW Calgary, Canada; 4 Department of Community Health Sciences. University of Calgary, 3330 Hospital Drive NW, Calgary, Canada

**Keywords:** Depressive Disorder, Epidemiologic Methods, Markov Chain

## Abstract

**Background:**

Most epidemiological studies of major depression report period prevalence estimates. These are of limited utility in characterizing the longitudinal epidemiology of this condition. Markov models provide a methodological framework for increasing the utility of epidemiological data. Markov models relating incidence and recovery to major depression prevalence have been described in a series of prior papers. In this paper, the models are extended to describe the longitudinal course of the disorder.

**Methods:**

Data from three national surveys conducted by the Canadian national statistical agency (Statistics Canada) were used in this analysis. These data were integrated using a Markov model. Incidence, recurrence and recovery were represented as weekly transition probabilities. Model parameters were calibrated to the survey estimates.

**Results:**

The population was divided into three categories: low, moderate and high recurrence groups. The size of each category was approximated using lifetime data from a study using the WHO Mental Health Composite International Diagnostic Interview (WMH-CIDI). Consistent with previous work, transition probabilities reflecting recovery were high in the initial weeks of the episodes, and declined by a fixed proportion with each passing week.

**Conclusion:**

Markov models provide a framework for integrating psychiatric epidemiological data. Previous studies have illustrated the utility of Markov models for decomposing prevalence into its various determinants: incidence, recovery and mortality. This study extends the Markov approach by distinguishing several recurrence categories.

## Introduction

In a series of previous reports, we have described the use of Markov models in major depression epidemiology. In two initial papers, we described a general approach to modeling, in which prevalence was depicted as a steady state outcome of inflow and outflow from a prevalence pool [1,2]. Subsequently, the approach was extended to include strata for age and sex categories [3], other demographic variables [4] and chronic conditions [5]. Broadly speaking, Markov models are useful for medical decision modeling and economic analyses. In the case of major depression, many of the most important health policy decisions relate to the longitudinal course of the condition. Clinical practice guidelines, for example, frequently distinguish between high recurrence and low recurrence groups, subjects with a high risk of recurrence being candidates for long-term treatment. In this report, we describe an application of Markov modeling to description of the longitudinal course of major depression.

Markov models are important in this context because the current literature presents few other options for modeling. To our knowledge, the only other example of an epidemiological general population model is that reported by Kruijshaar et al. [6] using microsimulation. This study integrated data from European (NEMESIS) [7] and Australian studies [8]. The use of more than one data source in this study illustrates the potential usefulness of modeling as a means of integrating the best available epidemiological data into a coherent epidemiological description. For purposes such as surveillance, policy development and cost-effectiveness analysis, data integration using epidemiological modeling is a promising approach.

Markov models, also known as health state transition models, divide a target population into a series of mutually exclusive health states. Transitions between these health states are assigned probabilities and the model's predictions are evaluated over a series of stages [9].

## Data Sources

In a series of previous reports, we used data from a Canadian study called the National Population Health Survey (for additional information about the NPHS; see, ) to model the relationship between prevalence, incidence and mortality. The longitudinal component of the NPHS uses a nationally representative probability sample initially consisting of 17,262 subjects who have subsequently been followed through four biannual data collection cycles. The NPHS provides a valuable source of longitudinal health data. A limitation, however, is that the NPHS utilized only a brief predictive instrument for major depression, the Composite International Diagnostic Interview Short Form [10] for Major Depression (CIDI-SFMD). This instrument identifies subjects with a high probability of having met DSM-IV [11] criteria for major depression in the preceding year, but does not provide fully specified data about recurrence. The CIDI-SFMD instrument covers only one half (12 months) of the interval between NPHS interviews (conducted 24 months apart). The CIDI Short Form only covers the final 12 months of this 24-month interval. The proportion of subjects without major depression at one interview who have an episode at the next interview can be directly estimated from the NPHS data, but this estimate provides only an approximation of the annual incidence proportion.

The CIDI-SFMD also includes an item that assesses episode duration. This item elicits the number of weeks during the previous year that a subject with CIDI-SFMD major depression was depressed. Notably, the number of weeks depressed during the preceding year only precisely assesses episode duration in subjects whose episode began and ended within that year. A final limitation of the NPHS is its sample size. Although the longitudinal cohort was relatively large, the number of subjects with major depressive episodes during any particular interview cycle was small enough to result in appreciable imprecision in estimating the weeks depressed in the past year variable. Fortunately, Statistics Canada conducted a related survey, called the Canadian Community Health Survey (CCHS 1.1)  during the same time frame and using the same sampling frame and which also included the CIDI-SFMD. The CCHS 1.1 had a sample size of 130,880 subjects, and therefore offered much greater precision for estimating episode duration.

In order to estimate the proportion of the population falling into various recurrence categories, neither of the two data sources listed above were adequate. The NPHS, as noted, provides incomplete longitudinal follow-up and the CCHS 1.1 was strictly cross-sectional. In Canada, the best source of lifetime data is the Canadian Study of Mental Health and Wellbeing, also known as the CCHS 2.1 . This study used the same sampling frame as the other two, but evaluated major depression using the World Health Organization's Mental Health (WMH2000) project WMH-CIDI [12]. This instrument provided a basis for dividing the population into three broad categories of recurrence risk based on the pattern of recurrence reported by the CIDI. Subjects with no prior episodes of major depression (low risk of developing an episode), those having had one episode (moderate risk) and with multiple prior episodes (high risk).

## Approach to Markov Modeling

The Markov models developed here adopted the general format of an incidence-prevalence model, modeling the "prevalence pool" [13] for major depression as a function of the inflow to the pool (incidence and recurrence) and the outflow through recovery. For simplicity, mortality was not included in the models presented here since our previous work indicated that this variable did not have an important influence on the epidemiological dynamics [1]. Changes in health state (depressed and not-depressed) were evaluated over a series of one week stages. To deal with declining probabilities of recovery with mounting episode length, a Markov tunnel [9] was used to depict the process of recovery. The Markov tunnel is a way of adding flexibility to a Markov model. The Markov tunnel used in this model consisted of a series of depressed health states, so that at the onset of an episode (by definition at 2-weeks after the onset of symptoms) the subject occupies a health state that represents the first week in an episode. At the next stage, there can be a transition either to the non-depressed state or, alternatively, the subject can progress into a health state representing the next week of the episode. By selecting transitions back to the non-depressed health state that decline with each stage in the tunnel, the pattern of recovery can be flexibly depicted.

**Figure 1:**
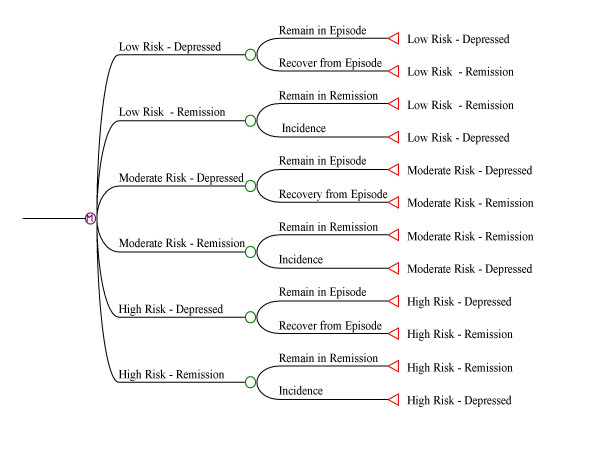
Framework for the Markov Model Employed in the Project, Depicting Three Recurrence Strata.

As noted above, it was not possible to directly estimate incidence and episode duration from the available data sources. Using tracking variables in the Markov model, it was, however, possible to define variables depicting parameters that are directly estimable: the proportion without an episode at one interview with an episode in the year prior to a subsequent interview and weeks depressed in the past year. The Markov models could then be evaluated by Monte Carlo simulation across a series of possible values for incidence and recovery to find the values most consistent with the observed estimates. The model is presented in Figure 1. The population is depicted in three strata, with size of the strata fixed according to the CCHS 1.2 results.

## Estimation from the Epidemiological Data Sources

Data from the three source studies were employed as described above: the NPHS to calibrate the transition probabilities for episode incidence, the CCHS 1.1 for weeks depressed in past year data and the CCHS 1.2 for the lifetime recurrence pattern. The sample sizes for the three studies were: 17,262 (at baseline), 130,880 and 36,525, respectively. Each survey used the same complex sampling frame. All reported estimates incorporated sampling weights and appropriate statistical procedures to deal with the resulting design effects. The estimates were made using SAS Version 8.1.

## Monte Carlo Simulation

The simulation period was set at 312 weeks (i.e. 6 years) in order to depict the duration of data available from the NPHS (data from the first four cycles, 1994 to 2000, had been released at the time when the analysis was conducted). Each simulation used 50,000 Monte Carlo trials to reduce random variation in the simulation output. Tracker variables were used to link the model output to directly estimable parameters, as described above.

A single Markov tunnel was used to describe the recovery pattern in each of the three incidence/recurrence risk categories. Essentially, this represents an assumption that episode prognosis is similar in the various recurrence categories. Successive Monte Carlo simulations were used to identify values for weekly incidence within each category that could explain both the observed overall incidence and duration data, as well as the pattern of recurrence observed over the six year NPHS follow-up period (the proportions of subjects having one, two or three detected episodes).

## Results

In the NPHS, the estimated proportion of the population without major depression at baseline who had one or more episodes during the subsequent six years (keeping in mind that the three interviews conducted during the six year follow-up period only covered three of these six years) was 9.3%. Of this population, 7.8% had one episode, 1.3% had two episodes and 0.3% had three episodes (these add to 9.4% due to approximation in rounding). The latter group represented those subjects who were positive on the CIDI-SFMD at each of the three follow-up visits. Some of these CIDI-SFMD positive instances may have represented persistence rather than recurrence, a distinction that could not be made using the NPHS data set.

According to data from the CCHS 1.2, the lifetime prevalence of major depression in the Canadian general population is 12.2% (95% C.I. 11.7% – 12.7%), consistent with European estimates [14] and somewhat less than American [15] estimates using the WMH2000 CIDI instrument. In the NPHS, 12.5% of the subjects had an episode at one or more of the follow-up interviews.

**Figure 2:**
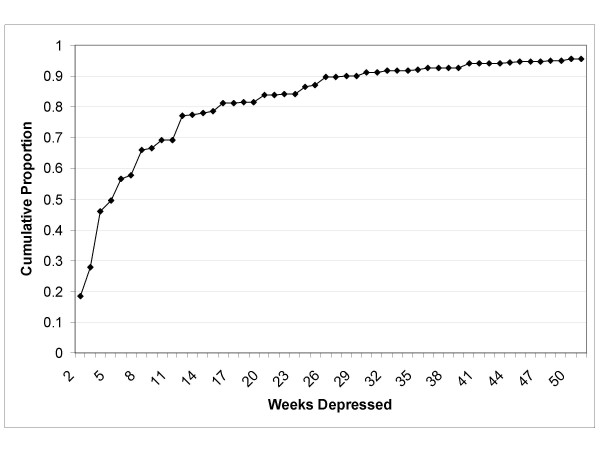
Cumulative Distribution of Reported Number of Weeks Depressed in Past Year, Subjects with CIDI-SFMD Major Depressive Episode.

The pattern for "number of weeks depressed" for subjects reporting an episode of major depression is presented as a cumulative proportion in Figure 2. Consistent with existing literature, many subjects had brief episodes, but as expected, the pattern suggested a declining probability of recovery with increasing episode duration. For example, the difference between the proportion of the population reporting *n *weeks depressed in the past year and *n*+1 weeks depressed in the past year became smaller as *n *became larger.

**Figure 3:**
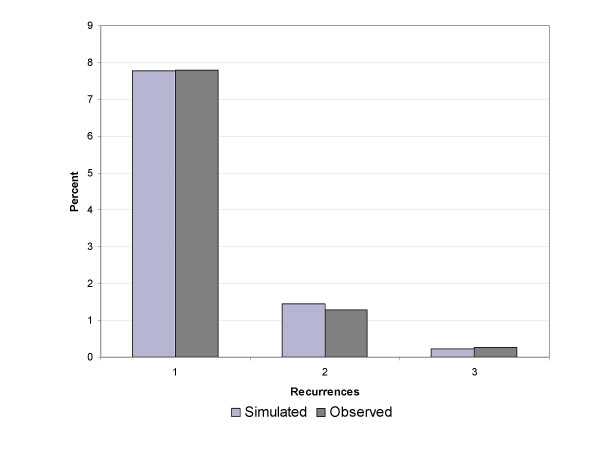
Observed and Simulated Proportions in NPHS Recurrence Categories.

**Figure 4:**
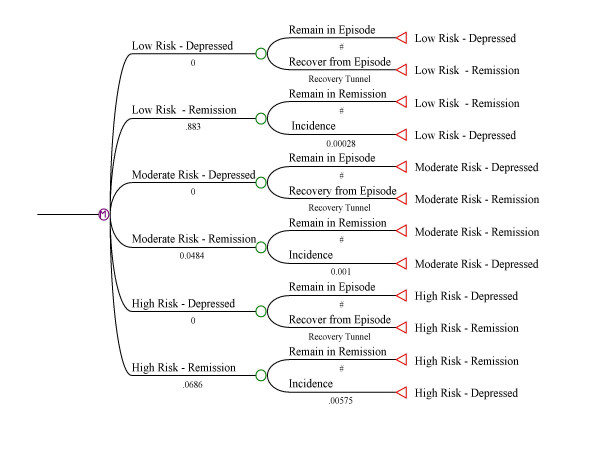
Specifications for the Markov Model.

As expected, the use of higher values for the incidence and recurrence resulted in a larger proportion of subjects with recurrent episodes. Because the proportions falling into the moderate and high recurrence groups were constrained by the CCHS 1.2 estimates, it was possible to identify, using a series of simulations, values for incidence within each recurrence category that resulted in the pattern observed in the NPHS. Figure 3 presents observed and simulated recurrence data for the parameters selected; Figure 4 is a depiction of the model, including values for the transition probabilities. Weekly transition probabilities were 0.00028 per week in the low risk, 0.0010 per week in the moderate risk and 0.00575 in the high risk categories.

**Figure 5:**
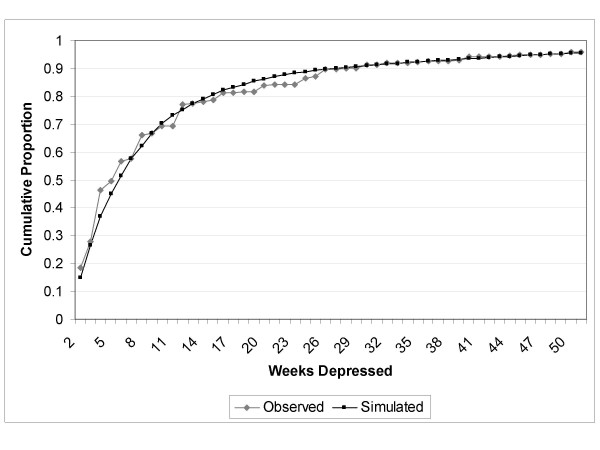
Observed and Simulated Weeks Depressed in the Past Year.

The Markov tunnel describing the weekly recovery probabilities (p_r_) followed the pattern of *p*_*r *_= 0.18*e^-0.09*week ^so that the probability of recovery was initially very high, but declined by approximately 9% with each week spent in the depressed state. The tunnel was truncated after 26 weeks, such that the probability of recovery once one half of a year was spent in the depressed state remained constant at approximately one half of one percent per week. Figure 5 juxtaposes the observed and simulated number of weeks depressed in the past year.

For ease of interpretation, it is possible to express the weekly transition probabilities as an expected annual cumulative incidence for depression using the formula: annual cumulative incidence = 1 - (1-weekly transition probability)^52^. Using this formula, the annual incidence in the low, moderate and high recurrence categories are: 1.4%, 5.1% and 25.9%. The weighted overall annual incidence is 3.3%, consistent with existing literature [16]. The mean number of weeks spent in the depressed state was estimated by Monte Carlo simulation in those with a single episode over the 312 week simulation interval, and was found to be 16.8 weeks (median = 9.0 weeks). These results are broadly consistent with the literature, where a range for mean duration of 12 to 30 weeks has been reported [17]. The considerable difference between median and mean duration has also been reported [18], and reflects the very long duration of a minority of episodes. Additional File 1 is the Markov model in the form of a Treeage^® ^Data Pro file, the Markov tunnel is contained in Additional File 2.

## Discussion

The model presented here projects a 9.3% overall new-episode frequency over a period of 6 years. Intuition suggests that since the duration of follow-up in the NPHS was essentially 6 years, lifetime prevalence should considerably exceed the observed occurrence of one or more episodes in the NPHS. This was not found to be the case, since lifetime prevalence from the CCHS 1.2, which used the WMH-CIDI was only 12.2%. This may be due to a lack of specificity of the CIDI-SFMD [19], which could bias the NPHS-derived incidence and prevalence estimates upwards. It is also possible that recall bias affected the WMH-CIDI lifetime prevalence estimate, which could bias the lifetime prevalence estimate downward. This interpretation is consistent with Kruijshaar et al.'s microsimulation results, which suggested that lifetime prevalence may actually be 20–30% [6] and is also consistent with the observation that approximately 50% of episodes are forgotten after a 25 year period [20]. Many previous authors have speculated that recall bias may result in underestimation of lifetime prevalence [21-23].

Since lifetime data from the WMH-CIDI were used to calibrate the Markov model presented here, and since the Kruijshaar et al. [6] microsimulation model suggested that recall bias may impact upon such estimates, the Markov model presented here may have been calibrated against an imperfect standard. Since recall bias would probably have the largest impact on remote episodes, the impact on our Markov model would probably be an underestimation of the proportion of the population in the low recurrence categories. In the future, studies with longer-term follow-up will be helpful for clarifying these dynamics more decisively.

Another limitation of these data was the assumption that the three categories identified by the CCHS 1.2 represented the proportion of the population falling into three broad recurrence categories. Subjects with no lifetime major depression were used to approximate the size of a low-risk category in the population, single episodes a moderate risk category and multiple episodes a high risk category. This division was somewhat arbitrary, but needed to be adopted because the NPHS data was also used to estimate incidence (it would have been tautological to estimate incidence from the same proportions that defined recurrence risk).

Figure 4 presents a more advanced Markov-based framework describing major depression epidemiology than previously described Markov models, and the model appears to provide a good description of available Canadian major depression data, despite the specified limitations. To our knowledge, such a method for estimating incidence and recurrence of major depression has not been previously reported. Markov models are commonly used in cost-effectiveness modeling (e.g. see, Sorensen et al. [24]). More recently, integration of data from psychiatric epidemiological surveys and clinical trials for cost-utility analyses has been described [25,26]. While available modeling approaches have limitations, they do provide a methodological framework which should support increasingly meaningful descriptions of major depression epidemiology. Of most importance, it should be possible to refine these models since they provide a platform for integrating the best available information as this becomes available.

## Disclaimer

The analyses reported here are based on data collected by Statistics Canada , but do not represent the opinions or interpretations of Statistics Canada.

## Supplementary Material

Additional File 1 This is the Markov model, as a Treeage^® ^Data Pro file. Click here for file

Additional File 2 The Markov model contains a Markov tunnel describing the pattern of recovery. This is the tunnel required – in the form of a table file for Treeage^® ^Data Pro. Click here for file
